# Co‐expression of NF‐κB‐p65 and phosphorylated NF‐κB‐p105 is associated with poor prognosis in surgically resectable non‐small cell lung cancer

**DOI:** 10.1111/jcmm.13476

**Published:** 2018-01-24

**Authors:** Gen Lin, Chao Li, Cheng Huang, Wu Zhuang, Yunjian Huang, Haipeng Xu, Qian Miao, Dan Hu

**Affiliations:** ^1^ Department of Thoracic Oncology Fujian Cancer Hospital & Fujian Medical University Cancer Hospital Fuzhou China; ^2^ Department of Pathology Fujian Cancer Hospital & Fujian Medical University Cancer Hospital Fuzhou China; ^3^ Fujian Provincial Key Laboratory of Translational Cancer Medicine Fuzhou China

**Keywords:** non‐small cell lung cancer, nuclear factor‐kappa b, p65, p105, prognosis

## Abstract

Nuclear factor‐kappa B (NF‐κB) as a prognostic marker remains unclear in non‐small cell lung cancer (NSCLC). Here, we studied NF‐κB‐p65 (p65) expression and phosphorylated NF‐κB‐p105 (p‐p105) expression in NSCLC and correlated the finding with overall survival (OS) and clinicopathological features. A total of 186 archival samples from patients with surgically resectable NSCLC were probed with p65 and p‐p105 (Ser 932). The p65‐positive expression and p‐p105‐positive expression were defined as distinct nuclear p65 and cytoplasmic p‐p105 labelling in at least 1% of tumour cells, respectively. The positive staining of p65 alone, p‐p105 alone and co‐expression of p65 and p‐p105 were observed in 61 (32.8%), 90 (48.4%) and 35 (18.8%) patients, respectively. Co‐expression of p65 and p‐p105 but not of either p65 or p‐p105 alone was associated with a poor prognosis. Patients with co‐expression of p65 and p‐p105 had a shorter OS than others, median OS 26.5 months *versus* 64.1 months, HR 1.85 (95% CI: 1.18–2.91), *P* = 0.007. There was no statistically significant association between clinicopathological characteristics and either p65 or p‐p105 alone or co‐expression of p65 and p‐p105. This indicates that co‐expression of p65 and p‐p105 was a poor prognostic factor, and pathologic studies of NF‐κB expression could include multiple pathway components in NSCLC.

## Introduction

Nuclear factor‐kappa B (NF‐κB) was initially discovered as a transcription factor in the nucleus of B cells that binds to the enhancer of the immunoglobulin kappa light chain gene, and has since been shown to be expressed ubiquitously in the cytoplasm of all types of cells. NF‐κB transcription complexes form a variety of homo‐ and heterodimers consisting of the subunits NF‐κB1 (p50 and its precursor p105), NF‐κB2 (p52 and its precursor p100), RelA (p65), RelB and c‐Rel. Through various combinations of subunits, the Rel protein family members can form up to 15 different dimers. Among them, the p50/65 heterodimer is the most abundant of Rel dimer, being found in almost all cell types 1, 2.

The NF‐κB signalling pathways can be divided into canonical and non‐canonical pathways. In the canonical pathway, I kappa B kinase (IKK) phosphorylates I kappa Bα (IκBα) at two N‐terminal serines, triggering its ubiquitination and proteasomal degradation. This leads to the nuclear translocation of the NF‐κB complexes, predominantly p50/p65 and p50/c‐Rel dimers. The non‐canonical NF‐κB pathway involves different signalling molecules and leads to the activation of the p52/RelB dimer 1, 2.

A large body of *in vitro* evidence supports NF‐κB as an important player in the development and progression of malignant cancers. NF‐κB targets genes that promote tumour cell proliferation, survival, metastasis, inflammation, invasion, angiogenesis and resistance to chemo‐ and radiotherapy 2. Constitutive or aberrant activation of NF‐κB is frequently encountered in lung cancer 3, 4, 5. However, a limited number of clinical studies had reported various degree of NF‐κB expression detected by IHC in lung cancers ranging from about 10% to 67%, reviewed by Wu 6. The same series of studies have correlated NF‐κB expression with clinicopathological characteristics and survival, but the results have been inconsistent. In addition, in most studies, pathologic studies of NF‐κB expression were detected by assaying for single components of the NF‐κB pathway, and using different anti‐NF‐κB subunit antibody clones, judgement criteria and cut‐off values for assessing NF‐κB expression were used in the various studies 6, 7, 8, 9, 10, 11, 12, 13, 14, 15. Actually, different NF‐κB dimers may have different functions, especially the p50‐p50 homodimer, which lacks a transactivation domain in the C‐termini and has no intrinsic ability to activate transcription. There is evidence that the p50‐p50 homodimer can act as a transcriptional repressor when binding κB elements 1, 16, 17, 18.

To address the above issues, we investigated the expression of p65 and p‐p105 in patients with surgically resectable NSCLC and the association between clinicopathological characteristics, overall survival and the expression of p65 and p‐p105.

## Patients and methods

### Patients

Primary tumour samples were from archives of patients with surgically resectable and pathological confirmed NSCLC at the Fujian Cancer Hospital in China between January 2008 and December 2010. None of the patient had prior anticancer therapies. The clinicopathological information of patients was collected from the clinical records and pathology reports. The pathological TNM stage was reassigned according to the 8th TNM staging 19, and lung tumour histology was reclassified according to the 2015 World Health Organization (WHO) classification for lung tumours 20. The study design was approved by the Ethical Committee of Fujian Cancer Hospital, and written informed consent was obtained from all patients (Number: SQ2017‐015‐01).

### NF‐κB‐p65 and p‐p105 immunohistochemistry

Immunohistochemistry detection of NF‐κB‐p65 (D14E12, Code #8242, CST) and p‐NF‐κB‐p105 (Rabbit monoclonal against Ser933, Code 178F3, CST) was carried out as previously described 21, 22. NF‐κB‐p65 activation in tumour cells was determined by distinct nuclear immunostaining in at least 1% of tumour cells. NF‐κB‐p105 activation in tumour cells was defined by distinct membranous or cytoplasmic immunostaining in at least 1% of tumour cells. Each assay contained positive and negative controls along with a negative isotype‐matched antibody control for each sample. Two board‐certified pathologists (CL and DH) independently evaluated all stained slides. The discordant cases were reviewed to reach a final consensus classification.

### Statistical analysis

Expression of p65, p‐p105 and co‐expression of p65 and p‐p105 were compared in subgroups based on age, gender, smoking status, histology, TNM stage or lymphatic vascular invasion using the binary logistic analysis. Adjustments were made for above‐mentioned factors in multivariate binary logistic analysis.

Overall survival (OS) was defined as the time from the date of diagnosis to the date of death or last follow‐up. The Kaplan–Meier method and a log‐rank test were used for univariate survival analysis. Survival rate correlation with age, gender, smoking status, histology, TNM stage, p65 expression, p‐p105 expression and co‐expression of p65 and p‐p105 was estimated by the Kaplan–Meier method, and survival curves were compared with the log‐rank test. Cox proportional hazard models were used for multivariate survival analysis that controlled for the above factors in the univariate survival analysis, and the hazard ratio (HR) and 95% confidence interval (CI) were estimated.

Statistical analyses were performed using SPSS 16.0 software (SPSS China, China). All tests were two‐sided. Statistical significance was set at *P* < 0.05.

## Results

### Patient characteristics

A total of 186 patients were eligible for the study, and their characteristics were summarized in Table 1. Median age at diagnosis was 55 years (range, 34–78 years), and the ECOG performance status was 0 in all patients. Adenocarcinoma and squamous carcinoma were the major histologic subtypes, accounting for 97 of 186 (55.3%) and 75 of 186 (40.3%), respectively. In this study, we also collected 11 adenosquamous carcinoma and other uncommon pathological types including one sarcomatoid carcinoma, one mucoepidermoid cancer and one lymphoepithelioma‐like cancer (Table 1).

**Table 1:** Association between p65 expression, p‐p105 expression and clinicopathological characteristics

	Total *N* (%)	p65		*P*	p‐p105		*P*	Co‐expression of p65 + p‐p105		*P*
		Negative (%)	Positive (%)		Negative (%)	Positive (%)		Negative (%)	Positive (%)	
Age										
>60 years	78 (41.9)	57 (45.6)	21 (34.4)	0.158	38 (39.6	40 (44.4)	0.502	68 (45.0)	10 (28.6)	0.075
≤60 years	108 (58.1)	68 (54.4)	40 (65.6)		58 (60.4)	50 (55.6)		83 (55.0)	25 (71.4)	
Sex										
Male	130 (69.9)	82 (65.6)	48 (78.7)	0.088	65 (67.7)	65 (72.2)	0.526	103 (68.2)	27 (77.1)	0.318
Female	56 (30.1)	43 (34.4)	13 (21.3)		31 (32.3)	25 (27.8)		48 (31.8)	8 (22.9)	
Smoking										
Never smokers	105 (56.5)	73 (58.4)	32 (52.5)	0.529	53 (55.2)	52 (57.8)	0.768	86 (57.0)	19 (54.3)	0.851
Former or current smokers	81 (43.5)	52 (41.6)	29 (47.5)		43 (44.8)	38 (42.2)		65 (43.0)	16 (45.7)	
Histology										
Ad	97 (52.2)	65 (52.0)	32 (52.5)	1.000	52 (54.2)	45 (50.0)	0.660	79 (52.3)	18 (51.4)	1.000
Non‐Ad	89 (47.8)	60 (48.0)	29 (47.5)		44 (45.8)	45 (50.0)		72 (47.7)	17 (48.6)	
Lymphatic vascular invasion										
Absence	164 (88.2)	113 (90.4)	51 (83.6)	0.226	86 (89.6)	78 (86.7)	0.538	136 (90.1)	28 (80.0)	0.141
Presence	22 (11.8)	12 (9.6)	10 (16.4)		10 (10.4)	12 (13.3)		15 (9.9)	7 (20.0)	
TNM stage										
I	54 (29.0)	38 (30.4)	16 (26.2)	0.723	29 (30.2)	25 (27.8)	0.454	45 (29.8)	9 (25.7)	0.567
II	40 (21.5)	25 (20.0)	15 (24.6)		17 (17.7)	23 (25.6)		30 (19.9)	10 (28.6)	
III	92 (49.5)	62 (49.6)	30 (49.2)		50 (52.1)	42 (46.7)		76 (50.3)	16 (45.7)	

### The p65 expression and p‐p105 expression and correlation with clinicopathological characteristics

**Figure 1:**
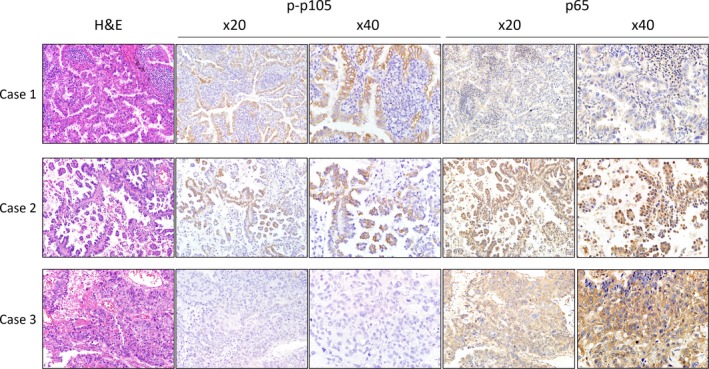
Immunohistochemical staining for p65 and p‐p105 in representative tissue specimens. Case 1 represented positive p‐p105 and negative nuclear p65. Case 2 represented positive p‐p105 and positive nuclear p65. Case 3 represented negative p‐p105, and negative nuclear but positive cytoplasmic p65.

**Figure 2:**
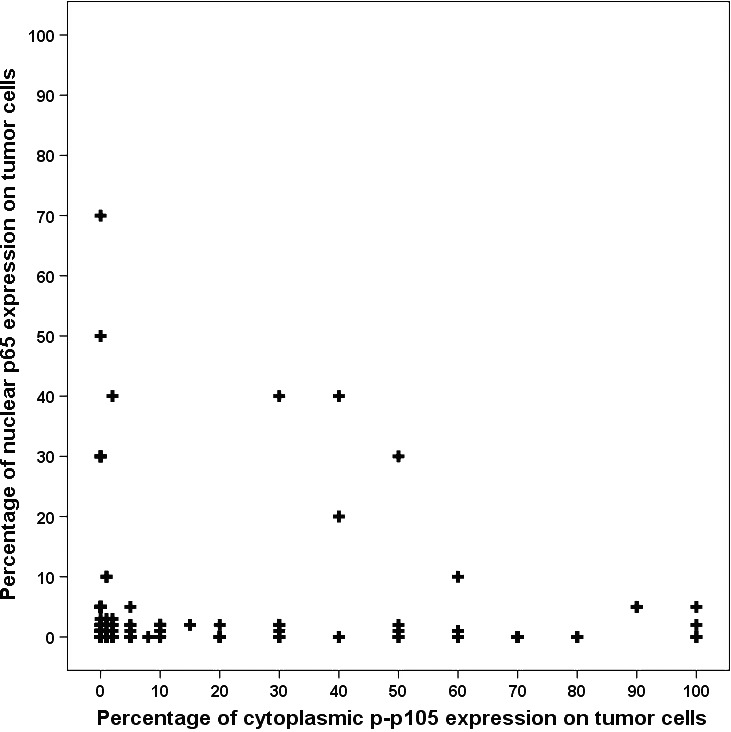
Scatter plot did not reveal a linear relationship between p65 expression and p‐p105 expression.

We first examined p65 expression and p‐p105 expression in normal lung areas adjacent to surgically resected tumour samples. In bronchial and alveolar epithelium, p65 exhibited low or moderate cytoplasmic expression. In tumours, both of p65 and p‐p105 subunits were highly expressed relative to normal areas. The p65 displayed both a nuclear and cytoplasmic expression patterns in areas of tumour cells. However, p‐p105 nuclear immunostaining was seldom observed (only in three patients) in tumour areas. In addition, p‐p105 expression showed more uniform distribution than p65 expression, which was dispersed within the tumour area in some cases. Representative images of p65 and p‐p105 IHC staining are shown in Figure 1. Furthermore, in our study, there was no linear relationship between p65 expression and p‐p50 expression in tumour cells, and p65 expression and p‐p50 expression were highly discordant in some cases and show a near mutual exclusion (Fig. 2).

The p65‐positive staining and p‐p105‐positive staining were observed in 61 of 186 (32.8%) and 90 of 186 (48.4%) of the patients’ tumours, respectively. In the present study, we found 35 (18.8%) tumours with co‐expression of p65 and p‐p105, 26 (14.0%) with p65‐positive expression alone, 55 (29.6%) with p‐p105‐positive expression alone and 70 (37.6%) that were both p65 negative and p‐p105 negative in this population.

The co‐expression of p65 and p‐p105 or the expression of either p65 or p‐p105 alone was correlated with the clinicopathological characteristics by univariate analysis (Table 1). There was no statistically significant association between co‐expression of p65 and p‐p105 or p65/p‐p105 alone and gender, age, smoking status, histology, lymphatic vascular invasion and TNM stage in the univariate analysis (Table 1).

### Prognostic value of p65 expression and p‐p105 expression

Survival data in this study were censored on 07 January 2017. The median follow‐up time was 48.9 months (m) (range: 1.1–104.5 m), and 104 patients had cancer‐related death.

**Figure 3:**
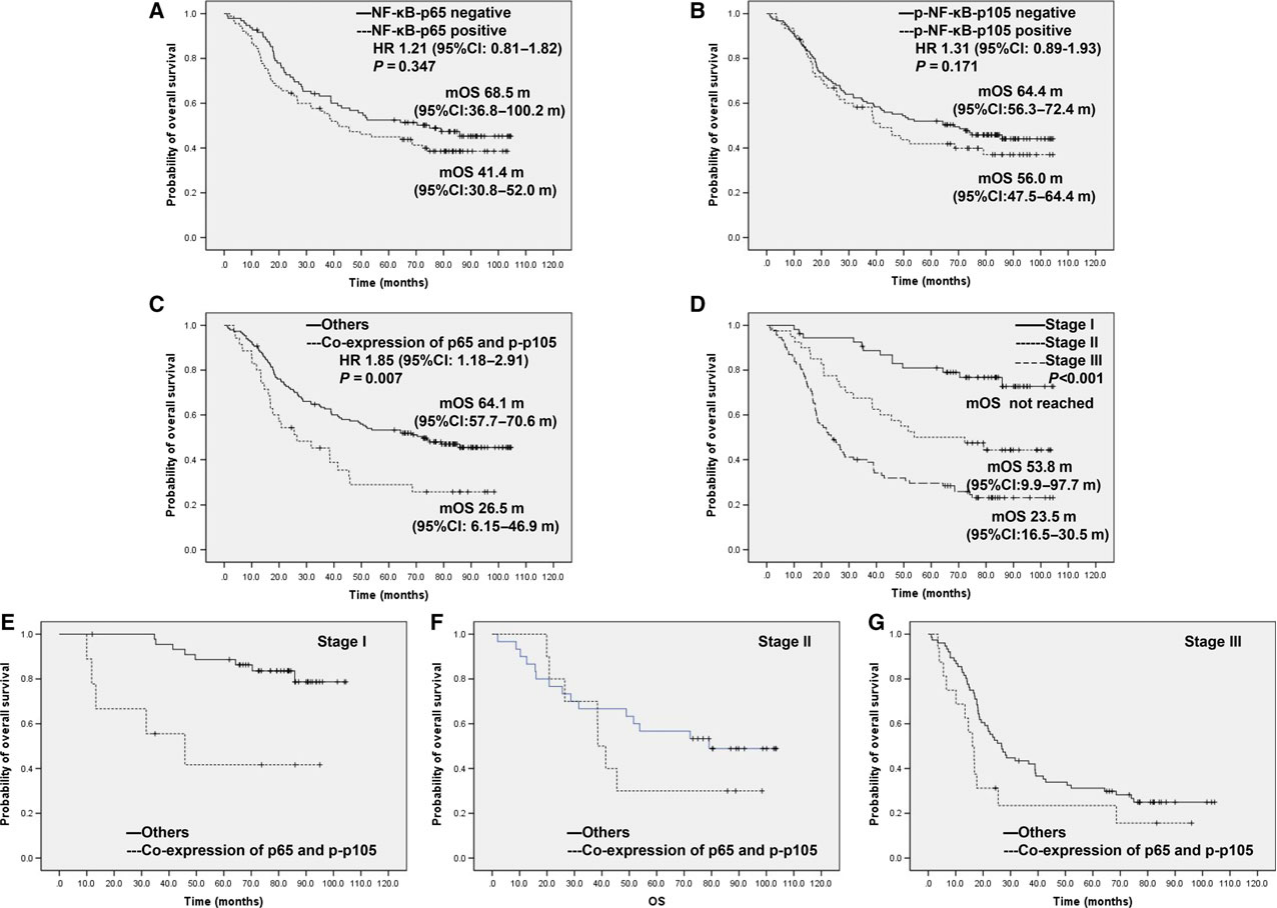
Prognostic value of p65, p‐p105 and co‐expression of them in the univariate survival analysis. **(A)** for p65 expression and OS. **(B)** for p‐p105 and OS. **(C)** for co‐expression of p65 and p‐p105 and OS. **(D)** for TNM stage and OS. **(E)** for co‐expression of p65 and p‐p105 in patients with Stage I and OS. **(F)** for co‐expression of p65 and p‐p105 in patients with Stage II and OS. **(G)** for co‐expression of p65 and p‐p105 in patients with Stage III and OS.

The unadjusted survival curves show a statistically significant association between TNM stage, lymphatic vascular invasion, co‐expression of p65 and p‐p105 and survival. Patients with co‐expression of p65 and p‐p105 had a shorter OS than others, median OS 26.5 months (95% CI: 6.15–46.9) *versus* 64.1 months (95% CI: 57.7–70.6), HR 1.85 (95% CI: 1.18–2.91), *P* = 0.007 (Fig. 3, Table 2). However, neither expression of p65 alone nor expression of p‐p105 alone was an independent prognostic factor.

**Table 2:** Univariate and multivariate analyses of OS in all patients

Variables	Reference	Univariate analyses		Multivariate analyses	
		HR(95% CI)	*P* value	HR(95% CI)	*P* value
Gender	Male	0.90 (0.59–1.38)	0.629		
Age	≤60 years	1.28 (0.87–1.88)	0.212	1.59 (1.06–2.38)	0.025
Histology	Squamous	0.84 (0.57–1.23)	0.373		
Smoking status	Never smokers	1.28 (0.87–1.88)	0.210	1.69 (1.13–2.53)	0.001
Lymphatic vascular invasion	Absence	2.71 (1.62–4.54)	**<0.001**	1.99 (1.17–3.38)	0.011
TNM stage	I (reference)		**<0.001**		<0.001
	II	2.75 (1.38–5.46)	**0.004**	2.26 (1.12–4.57)	0.023
	III	5.53 (3.05–10.05)	**<0.001**	5.84 (3.18–10.71)	<0.001
p65 expression	Negative	1.21 (0.81–1.82)	0.347		
p‐p105 expression	Negative	1.31 (0.89–1.93)	0.171		
Co‐expression of p65 and p‐p105	Negative	1.85 (1.18–2.91)	**0.007**	2.31 (1.42–3.75)	0.001

CI, confidence interval. The bold values represented statistical significance (*P* < 0.05).

Setting TNM stage as a stratification factor (Stage I *versus* Stage II *versus* Stage III), co‐expression of p65 and p‐p105 was still associated with overall survival in the population, *P* = 0.003 (Fig. 3E–G). In the multivariate Cox regression analysis, tumour stage, co‐expression of p65 and p‐p105, age, smoking status and lymphatic vascular invasion were shown to be significantly associated with OS after controlling for covariates (Table 2).

## Discussion

In the NF‐κB pathway, the p50/65 heterodimer obviously represents the most abundant of Rel dimmers. In this study, we studied expression of p65 and p‐p105 (precursor p50) in 186 patients with resectable NSCLC and found that co‐expression of p65 and p‐p105 but not p65 or p‐p105 alone was an independent prognosis biomarker. To our knowledge, this is the first attempt to link both p65 and p‐p105 with the outcome of NSCLC patients.

We noticed that some clinical studies reporting NF‐κB expression detected by IHC that they correlated with clinicopathological characteristics and/or survival in lung cancers had conflicting results; summarized in Table 3 7, 8, 9, 10, 11, 12, 13, 14, 15. The Rel protein family members can form up to 15 different dimers through combinatorial associations, and various antibodies for different key NF‐κB pathway components, such as p65, p105, IκBα and IKKα/β, were used for the evaluation of NF‐κB expression in the different studies 6. One of interpretation of discrepancy between study results could be different NF‐κB subunit antibodies and judgement criteria when comparing the experimental positives used in the different studies.

**Table 3:** Summary of studies investigating NF‐κB expression in NSCLC

Reference	Country	*N*	Marker	NF‐κB (+)%	Antibody	Cut‐off	Clinicopathological variables	Prognosis
Zhang *et al*.^15^	China	45	p50‐N/C	67.6	NR	≥10% tumour cells	Lower degree of differentiation;	Shorter OS
Qin *et al*. 7	China	115	RelB‐N/C	52.0	NR	>70% moderate staining or >10% strong staining	Lower degree of differentiation; Advanced TNM stage	Shorter OS
Tang *et al*. 12	USA	370	p65‐N	56.6	NR	≥50% tumour cells	Advanced TNM stage; KRAS/EGFR mutation	NS
Zhang *et al*. 11	China	116	p65‐N	48.3	NR	>5% tumour cells	Advanced TNM stage	NS
			p‐IκBα‐N/C	32.8	NR	>5% tumour cells	Adenocarcinoma;	NS
			p65 + p‐IκBα	18.1			Advanced TNM stage; Adenocarcinoma; Smoking status≥ 27 pack‐years	Shorter OS
Jin *et al*. 10	China	88	p65‐N	46.6	sc109	>5% tumour cells	Age≥55.1 year; Smoker; Advanced TNM stage;	Shorter OS
			p‐IκBα‐C	30.7	sc‐8404	>5% tumour cells	Adenocarcinomas; Advanced T stage;	Shorter OS
			p65 + p‐IκBα	18.1			Adenocarcinomas; Smoker; Advanced TNM stage; Advanced T stage;	Shorter OS
			p‐IKKα/β‐C	30.7	#2697S	>5% tumour cells	NS	NS
Al‐Saad *et al*. 14	Norway	335	p‐p105‐C	10.0	178F3	Scored by intensity ≥2	NS	Longer DSS
Zhang *et al*. 13	China	106	p65‐N/C	45.3	NR	≥10% tumour cells	Non‐squamous cancers; Node metastasis; Lower degree of differentiation;	NA
Nair *et al*. 9	USA	355	p65‐C	57.19	D14E12	>20% tumour cells	Increasing FDG uptake levels; Non‐adenocarcinomas; Advanced TNM stage	NS
GiopanouI *et al*. 8	German	79	p65‐N/C	31.6	sc‐8008	Scored by the intensity and distribution: low, intermediate and high	Men; Age ≥60 year; Squamous cancers	NA
			RelB‐N/C	13.9	sc‐226		Nodal involvement	
			p50‐N/C	38.0	sc‐114		Poor differentiation; Advanced TNM stage	
			p100/52‐N/C	36.7	ab31409		Advanced TNM stage	

N, nuclear expression; C, cytoplasmic expression; NR, not report; NS, not significant; NA, not available; OS, overall survival; DSS, disease‐specific survival.

Of those NF‐κB pathway components, p65 has been the most studied in the field of lung cancer. Recently, a systematic review showed that expression of NF‐κB, which was mainly focused on p65, was associated with worse survival in most solid tumours 6. However, two large studies found that p65 expression was not associated with overall survival in NSCLC irrespective of NF‐κB localization, which is consistent with our finding 9, 12. From a mechanistic perspective, nuclear expression is considered an active marker of NF‐κB, whereas cytoplasmic localization of NF‐κB is generally thought to indicate inactivation of the pathway. However, the situation is actually more complex. The crystal structure of IκBα when bound to the p65/p50 heterodimer reveals that the IκBα protein masks only the nuclear localization sequence (NLS) of p65, whereas the NLS of p50 remains exposed. The exposed NLS of p50 coupled with the nuclear export sequences in IκBα and p65 leads to constant shuttling of IκBα/NF‐κB complexes between the nucleus and the cytoplasm, despite steady‐state localization that appears almost exclusively cytosolic 1. Therefore, it is not completely reliable to justify NF‐κB pathway activation only by nuclear p65 expression.

There have been studies that attempted to evaluate the prognostic value of p105/p50 in NSCLC. A study, with a small sample size, found overexpression of p50 to indicate an unfavourable overall survival 15. However, in a study that included 335 patients with NSCLC, Al‐Saad *et al*. 14 found that p‐p105 expression, detected by assaying for p‐p105 (Ser933) (CST 178F3, Rabbit mAb), was a favourable independent prognostic indicators for survival. The IκB/NF‐κB precursor protein p105 undergoes processing *via* the proteasome to yield p50. Multiple reports have demonstrated that IKKβ‐dependent phosphorylation of the C‐terminal region of p105 at Ser933 (in human p105) leads to complete degradation of the protein 1, 17. In this study, we used the same antibody assay as the Al‐Saad study; however, our study did not confirm the above finding. Actually, the p50 proteins, which lack a transactivation domain in the C‐termini, have no intrinsic ability to activate transcription, and evidence showed that the p50‐p50 homodimer can act as transcriptional repressors when binding κB elements 1, 16, 17, 18. Therefore, it may also be unable to safely justify activate transcription by active p50 overexpression alone.

Taken together, the above findings strongly suggest pathologic studies of NF‐κB expression in NSCLC may need to include multiple pathway components. In this study, we found that co‐expression of p65 and p‐p105 but not p65 or p‐p105 alone was confirmed to be associated with a poor prognosis. Similar results were obtained in other studies. Two separate studies showed that composite application of multiple biomarkers (both nuclear p65 expression and p‐IκB‐α expression) independently predicts poorer prognosis in NSCLC patients 10, 11. Strikingly, co‐expression of p65 and p‐p105 was a strong predictor of overall survival, particularly in stage I, despite the limitation in the small number of patients with co‐expression of p65 and p‐p105. A prospective study in a larger population that will include early‐stage patients will be carried out in the future.

In conclusion, our study showed co‐expression of p65 and p‐p105 but not p65 or p‐p105 alone was a poor prognostic indicator of survival outcome in early‐stage NSCLC, which indicated pathologic studies of NF‐κB expression in NSCLC may need to include multiple pathway components.

## Conflict of interest

The authors confirm that there are no conflict of interests.
